# Socio-demographic factors, dental status and health-related behaviors associated with geriatric oral health-related quality of life in Southwestern China

**DOI:** 10.1186/s12955-018-0925-8

**Published:** 2018-05-21

**Authors:** Rui Shao, Tao Hu, Yi-Si Zhong, Xue Li, Yi-Bo Gao, Yi-Fan Wang, Wei Yin

**Affiliations:** 1 State Key Laboratory of Oral Diseases, National Clinical Research Center for Oral Diseases, Department of Preventive Dentistry, West China Hospital of Stomatology, Sichuan University, No. 14, Section 3, Renmin South Road, Chengdu, 610041 China; 2 Department of Stomatology, The General Hospital of the People’s Liberation Army, Beijing, China

**Keywords:** Oral health-related quality of life, Elders, GOHAI, China, Epidemiology

## Abstract

**Background:**

The aging of Chinese society has increased interest in improving the health-related quality of life (HRQoL) of the elderly, including their oral health-related quality of life (OHRQoL)**.** This study aims to evaluate the OHRQoL of elders living in Sichuan Province (China) and to explore the explanatory factors of their OHRQoL.

**Methods:**

A cross-sectional study conducted in 2016 in the Sichuan Province analyzed data from 744 elders, aged 65 to 74 years (mean age 69.3, 51.3% female). Clinical examinations and questionnaires were completed to collect information on the participants’ socio-demographic characteristics, health-related behaviors, dental status, subjective health conditions and General Oral Health Assessment Index (GOHAI) score.

**Results:**

The mean GOHAI score was 48.23 (SD 7.62), and the median score was 49. After adjustment for age and gender, the multiple linear regression analysis showed that participants who were female, had fair or poor self-rated oral health, decayed, missing and filled teeth (DMFT) score ≥ 20, fair or poor self-rated general health, and ≥ 2 teeth with root caries had worse OHRQoL, and participants who were edentulous had better OHRQoL (F = 29.58, *p* < 0.001).

**Conclusion:**

The OHRQoL of the elders living in Sichuan Province was relatively good. The explanatory variables were gender; self-rated oral health; DMFT score; self-rated general health; number of natural teeth; and number of teeth with root caries. More attention should be paid to caries status and retention of healthy teeth to improve the OHRQoL of elders in Sichuan Province, preserving a healthy mouth contributes to better OHRQoL.

## Background

China is the country with the largest elderly population in the world, it is generally accepted that China has already come into the aging society. In 2010, the number of people over the age of 65 years exceeded 111 million and accounted for 8.2% of the total population. It is predicted that in 2050 there will be a large explosion in the elderly population numbers, with up to 400 million people aged over 65, the percentage will increase to 26.9% [1]. Facing such a large elderly population, in terms of the health problems, not only their physical or psychological health but also their health-related quality of life has become a major public health concern [2].

Research has shown that oral health is an essential element of general health and that quality of life yet is often overlooked when integrated approaches to promote general health are created [3]. According to the report of the Third National Survey of Oral Health Status in China in 2005, the caries prevalence rate in elderly Chinese individuals, aged 65 to 74 years is increasing, and the majority of these adults display tooth loss and poor oral health status [4]. Oral disease in the elderly not only leads to pain, diminished masticatory ability, and adverse effects on pronunciation and physical appearance but also affects general health and increases the risk of neurological, endocrine, cardiovascular and respiratory disease. Oral health problems are associated with changes in food selection, decreased nutritional quality and decreased quality of life [5].

Oral health is perceived as a distinct dimension of overall quality of life [6], and oral health-related quality of life is subject to the comprehensive assessment of oral status in the areas of physical, psychological and social function. It is defined as the cyclical and self-renewing interaction between the relevance and impact of oral health [7].

Among related studies from the past twenty years, several instruments have been used to measure OHRQoL. The General Oral Health Assessment Index (GOHAI), one of the most commonly used instruments, was initially designed by American scholars in 1990 [8] to assess self-reported oral health status in elderly populations. Based on the responses to 12 questions, it evaluates OHRQoL with scores in three domains: physical function, psychosocial function and pain or discomfort. Currently, this instrument has been translated into various languages; a Chinese version (GOHAI-H) was developed in 2002 by Hong Kong scholars [9] and adapted for use in mainland China. It was translated into Mandarin Chinese the following year [10], and that version is commonly used in epidemiological studies. In the fifth version of the Oral Health Surveys published by the World Health Organization in 2013 [11], the GOHAI was recommended as the standard data collection method for analyzing OHRQoL and its impact factors.

Scholars and professionals in many countries have investigated the factors that impact the OHRQoL of the elderly. Japanese scholars proved that GOHAI scores were significantly lower for participants with poor perceived oral and general health, a low level of education, and a higher number of remaining teeth and for those who felt that they needed dental treatment and did not wear removable prostheses [12]. German researchers also found that denture status was a stronger predictor of impaired OHRQoL [13]. In addition, a study of an elderly Mexican population showed their GOHAI scores was associated with the responses regarding self-perceived oral and general health, the missing and filled components of the DMFT index and the number of healthy and functional teeth [14]. Moreover, some reports have evaluated the OHRQoL of Chinese elderly adults in different regions. Wang and Ling surveyed 263 Chinese elders in Guangdong Province in southern China and found that OHRQoL was associated with self-rated oral health, number of missing teeth and satisfaction with life [15]. Another report studied elders in Shandong Province in eastern China and concluded that OHRQoL was influenced by age and a number of clinical factors [16].

Using a deep analysis of the data from the epidemiological survey of oral health status in Sichuan Province in southwestern China, the main purposes of this study were to evaluate the OHRQoL of elders living in Sichuan Province; to investigate the association between socio-demographic factors, oral clinical factors, health-related behaviors factors, subjective health conditions and OHRQoL; and to explore the explanatory factors of OHRQoL in this population of Chinese elderly adults.

## Methods

### Population and sample

This report presents an analysis of the data from the epidemiological survey of oral health status in Sichuan Province conducted from December 2015 to May 2016 [17]. Sichuan Province is located in southwest China and is inhabited by multiple ethnic groups, with a population of 81.40 million. The economic aggregate of Sichuan ranks first in western China and sixth overall in China.

In this cross-sectional study, the subjects were permanent urban and rural residents living in Sichuan Province. The sample was chosen using a four-stage stratified random-cluster survey sampling method [18]. In the first stage, two districts and two counties were chosen randomly by stratified sampling using the probability-proportional-to-size (PPS) method. In addition, because these four areas represented middle and low urbanization levels, to obtain a sufficiently representative sample, another district and county were chosen by PPS in the second stage of sampling. Finally, six areas of Sichuan Province (Guang’an District, Chuan’shan District, Jin’niu District, Da County, Yi’bin County, and Pi County) were selected. In the third stage, the PPS method was used to select three neighborhood committees and three village committees in each of the six areas. In the fourth stage, the individuals who were interviewed in the selected communities were chosen using a quota sampling method that excluded people with serious physical or psychological illness or disadvantages and those who were unable or unwilling to finish the survey. Finally, a total of 744 persons aged 65 to 74 years responded to the invitation and completed the survey, which was more than the required sample size of 696 obtained with the following formula:

$$ n= deff\frac{\mu^2p\left(1-p\right)}{\varepsilon^2} $$

The design efficiency *deff* = 2.5, the level of confidence *μ* = 1.96, the margin of error ε = 10%, the prevalence of caries in 65- to 74-year age group in the third national epidemiological survey of oral health status was *p* = 86.0%, and the non-response rate was 10%.

### Measures

A standardized WHO survey instrument, the 5th version of Oral Health Surveys – Basic Methods [11], which includes modules on dentition status, periodontal status, dentures, oral mucosa, self-reports of oral health and risk factors, was used across all six areas. To explore the explanatory factors comprehensively in reference to oral health risk indicators within the STEPS framework recommended by WHO, except for oral clinical variables and subjective health conditions, we also included socio-demographic factors. In addition, we selected the health-related behaviors variables smoking habits, drinking habits, brushing habits and frequency of eating sweets to investigate their relationship with OHRQoL.

Items related to the experience of reduced quality of life due to oral problems (12 questions) were included among these modules, which are consistent with the GOHAI questionnaire translated by panel of the Chinese Stomatological Association according to the questionnaire recommended by the WHO [11]. Before its use in the present study, the Chinese questionnaire was pilot-tested in Ensi, Hubei Province, in July 2016 prior to the actual survey, and its reliability and validity were high [19, 20]. Trained investigators conducted the interview of questionnaire and prior to being surveyed, all subjects signed a consent form.

Three licensed dentists from the West China Hospital of Stomatology at Sichuan University were field trained; the mean kappa values used to determine inter-examiner reliability were > 0.80 for the periodontal examination and > 0.85 for the dental caries examination, and the intra-examiner reliability of each examiner was also > 0.80. Clinical evaluations were performed with the subject in a portable dental chair and using artificial light, a #5 dental mirror and a CPI probe. Infection control procedures were followed.

### Variables

To avoid human error, double entry and validation were used to enter the data, resulting in data tables that included the following variables: Socio-demographic variables, including age (65–69 or 70–74 years), gender (male or female), household registration type (nonagricultural or agricultural) and educational level (illiterate, low: primary school, medium: junior high school, high: high school or above); health-related behaviors variables, including smoking habits (smoking or not smoking), drinking habits (drinking or not drinking), brushing habits (seldom/never, less than once a day, once a day, twice a day or more), frequency of eating sweets (seldom/never, less than once a day, once a day or more); oral clinical variables, including DMFT (0–9, 10–19, ≥20), number of natural teeth (0, 1–8, 9–16, 17–24, ≥25), number of teeth with a periodontal pocket > 6 mm/deep pockets (0, 1, ≥2), number of teeth with loss of attachment (LOA) ≥6 mm (0, 1, ≥2), number of teeth with root caries (0, 1, ≥2), mucosa condition (normal or abnormal), number of teeth with unexposed root (0,1–16, 17–32), number of functional occluding pairs (occluding pairs that contain both natural teeth and bridge abutments, pontics) (0–9, 10–12, ≥13), whether the subject needs dentures but does not have them (no or yes) and presence and type of dentures (no dentures, fixed dentures, removable partial dentures, complete dentures and informal dentures, i.e., those made of self-curing resin and bent steel wire, which may damage to the patient’s oral tissue); and subjective health conditions, including self-rated general health (good or very good, fair, poor or very poor), self-rated oral health (good or very good, fair, poor or very poor).

### Analysis

The prevalence and mean values of all the variables were calculated. Bivariate analysis was performed using parametric tests (Student’s t test and ANOVA) to assess the association between each variable and the total GOHAI score. In addition, after screening out the variables that had statistically significant differences, a stepwise multiple linear regression analysis was conducted to establish significant associations between these variables and the GOHAI score. Because the Spearman rank correlation test showed that there were correlations between gender and smoking and drinking habits and between age and number of natural teeth and DMFT score, we enforced the gender and age variables into the multiple linear regression model first. The level of significance was set at 0.05 for all tests, and the analysis tool used was the statistics software IBM SPSS Statistics 20.0.

## Results

A total of 744 subjects were considered eligible for participation in this study. Among them, 51.3% were female, 54.3% had an agricultural household registration, and the mean age was 69.3 years. Two thirds of the elderly participants who were surveyed had obtained an educational level no higher than a primary school diploma, while 13.8% had finished high school.

The mean GOHAI score of the participants was 48.23 (SD 7.62); with a range from 19 to 60 and a median score of 49. Regarding the three domains of the GOHAI, the mean physical function score was 15.30 (SD 3.39), ranging from 5 to 20, with a median of 15; the mean pain and discomfort score was 11.35 (SD2.77), ranging from 3 to 15, with a median of 12; and the mean psychosocial function score was 21.61 (SD 3.50), ranging from 5 to 25, with a median of 22.

The parametric test results shown in Table 1 indicate that socio-demographic characteristics, such as being male, were significantly associated with higher GOHAI scores (*p* = 0.004). In addition, elders who were illiterate had lower GOHAI scores than those with a high educational level, and the differences were statistically significant (*p* = 0.001). All of the surveyed health-related behaviors factors shown in Table 2 except for the frequency of eating sweets had a statistically significant relationship with the GOHAI score. Elderly subjects who reported smoking (*p* < 0.01) or drinking (*p* = 0.03) had a higher GOHAI score. In addition, participants who seldom or never brushed their teeth had lower GOHAI scores than others (*p* < 0.03).

**Table 1:** Mean GOHAI score of the participants in relation to socio-demographic variables

Variables	n (%)	Mean GOHAI score ± SD	95% CI	*p*-value
Gender				0.004*
Male	362 (48.7)	49.07 ± 7.024	48.35, 49.80	
Female	382 (51.3)	47.47 ± 8.083	46.66, 48.28	
Household registration type				0.286*
Non-agricultural	340 (45.7)	48.63 ± 7.259	47.85, 49.40	
Agricultural	404 (54.3)	47.94 ± 7.912	47.16, 48.71	
Educational level				< 0.001**
Illiterate	123 (16.5)	45.80 ± 8.545	44.28, 47.33	
low	388 (52.2)	48.52 ± 7.677	47.75, 49.28	
Medium	128 (17.5)	48.20 ± 7.229	46.95, 49.45	
High	105 (13.8)	50.23 ± 5.903	50.53, 51.00	
Age				0.205*
65–69	414 (55.6)	47.93 ± 7.797	47.18, 48.69	
70–74	330 (44.4)	8.65 ± 7.392	47.85, 49.45	

*2-sample t-test

**One-way ANOVA

**Table 2:** Mean GOHAI score of the participants in relation to health-related behaviors variables

Variables	n (%)	Mean GOHAI score ± SD	95%CI	*p*-value
Smoking habits				0.004*
Smoking	260 (34.9)	49.34 ± 7.036	48.48, 50.20	
Not smoking	484 (65.1)	47.67 ± 7.866	46.96, 48.37	
Drinking habits				0.030*
Drinking	359 (48.3)	48.88 ± 7.476	48.10, 49.66	
Not drinking	385 (51.7)	47.66 ± 7.722	46.89,48.44	
Brushing habits				0.001**
Seldom/never	71 (9.5)	45.01 ± 9.086	42.86, 47.16	
Less than once a day	93 (12.5)	47.42 ± 8.065	45.76, 49.08	
Once a day	336 (45.2)	48.67 ± 7.180	47.90, 49.44	
Twice a day or more	244 (32.8)	48.93 ± 7.360	48.00, 49.86	
Frequency of eating sweets				0.797**
Seldom/never	218 (29.3)	48.14 ± 8.036	47.06, 49.21	
Less than once a day	407 (54.7)	48.41 ± 7.370	47.69, 49.13	
Once a day or more	119 (16.0)	47.92 ± 7.750	46.51, 49.32	

*2-sample t-test

**One-way ANOVA

**Table 3:** Mean GOHAI score of the participants in relation to oral clinical variables

Variables	n (%)	Mean GOHAI score ± SD	95% CI	*p*-value
DMFT				< 0.001**
0–9	355 (47.7)	49.72 ± 7.090	48.98, 50.46	
10–19	219 (29.4)	48.15 ± 7.233	47.18, 49.11	
≥ 20	179 (22.9)	45.32 ± 8.340	44.06, 46.59	
Number of natural teeth				< 0.001**
0	30 (4.03)	49.63 ± 7.609	46.79, 52.47	
1–8	31 (4.17)	45.68 ± 8.268	42.64, 48.71	
9–16	59 (7.93)	44.81 ± 8.953	42.48, 47.51	
17–24	141 (18.95)	46.60 ± 8.046	45.36, 47.94	
≥ 25	483 (64.92)	49.23 ± 7.045	48.60, 49.86	
Number of teeth with deep pockets				0.440**
0	580 (78.0)	48.37 ± 7.545	47.76, 48.99	
1	105 (14.1)	47.38 ± 7.808	45.87, 48.89	
≥ 2	59 (7.9)	48.61 ± 8.073	46.51, 50.71	
Number of teeth with LOA ≥ 6 mm				0.033**
0	384 (51.6)	48.96 ± 7.489	48.24, 49.68	
1	109 (14.7)	47.46 ± 7.231	46.09, 48.83	
≥ 2	251 (33.7)	47.51 ± 8.319	46.48, 48.55	
Number of teeth with root caries				< 0.001**
0	377 (50.7)	49.51 ± 7.489	48.75, 50.27	
1	123 (16.5)	48.25 ± 7.352	46.94, 49.56	
≥ 2	244 (32.8)	46.31 ± 7.586	45.35, 47.27	
Mucosa condition				0.090*
Normal	682 (91.7)	48.39 ± 7.600	47.82, 48.97	
Abnormal	62 (8.3)	46.68 ± 7.769	44.70, 48.65	
Number of teeth with unexposed root				0.466**
0	602 (80.9)	48.12 ± 7.610	47.51, 48.73	
1–16	121 (16.3)	48.63 ± 7.847	47.22, 50.04	
17–32	21 (2.8)	49.95 ± 6.689	46.91, 53.00	
Number of functional occluding pairs				< 0.001**
0–9	303 (40.7)	46.45 ± 8.173	45.52, 47.37	
10–12	135 (18.2)	8.88 ± 6.685	47.74, 50.02	
≥ 13	306 (41.1)	49.76 ± 7.079	48.96, 50.55	
Needs but does not have dentures				< 0.001*
No	425 (57.1)	49.14 ± 7.264	48.44, 49.83	
Yes	319 (42.9)	47.07 ± 7.937	46.20, 47.95	
Presence and type of dentures				0.132**
No dentures	358 (48.1)	48.32 ± 8.230	47.47, 49.18	
Fixed dentures	40 (5.4)	49.10 ± 4.667	47.61, 50.59	
Removable partial dentures	193 (25.9)	48.08 ± 7.188	47.06, 49.10	
Complete dentures	32 (4.3)	51.09 ± 6.260	48.84, 53.35	
Informal dentures	121 (16.3)	47.27 ± 7.420	45.94, 48.61	

*2-sample t-test

**One-way ANOVA

As Table 3 shows, nearly half of the elderly participants had fewer than ten decayed, filled or missing teeth; however, 22.8% had a DMFT score equal to or greater than twenty. In addition, 3.04% of the subjects were edentulous, 16.13% had ≤16 teeth present, and 64.92% of the subjects had more than twenty-two natural teeth. Among all the participants, approximately half had one or more teeth with root caries, 80.9% had exposed roots on all teeth, and 40.7% of them had fewer than ten functional occluding pairs.

Regarding denture status, almost half of the elderly participants used dentures, including 5.4% with fixed dentures, 25.9% with removable partial dentures, 4.3% with complete dentures and 16.3% with informal dentures. Moreover, 42.9% of the surveyed participants needed but did not have dentures. Regarding periodontal status, only 7.9% of the participants had more than two teeth with deep pockets, while 33.7% of them had more than two teeth with LOA ≥6 mm. In addition, 91.7% of the subjects had normal oral mucosa status. The parametric tests showed that among the oral clinical factors, DMFT score, number of natural teeth, number of teeth with root caries and functional occluding pairs were significantly associated with the GOHAI score (*p* < 0.001). Additionally, participants who did not have teeth with LOA ≥6 mm showed higher GOHAI scores, and the difference was statistically significant (*p* < 0.05).

Furthermore, in terms of the subjective health conditions shown in Table 4, 23.9% of the elderly participants rated their general health as poor or very poor, and 35.1% rated their oral health as poor or very poor. Those who rated their general health or oral health poor or very poor had lower GOHAI scores than the other participants (*p* < 0.001).

**Table 4:** Mean GOHAI scores of the participants in relation to subjective health conditions variables

Variables	n (%)	Mean GOHAI score ± SD	95%CI	*p*-value
Self-rated general health				< 0.001**
Good or very good	227 (30.5)	50.79 ± 6.482	49.95,51.63	
Fair	339 (45.6)	48.20 ± 7.649	47.38, 49.02	
Poor or very poor	178 (23.9)	45.11 ± 7.825	43.95, 46.26	
Self-rated oral health				< 0.001**
Good or very good	187 (25.1)	52.21 ± 6.307	51.30, 53.12	
Fair	296 (39.8)	45.96 ± 6.106	48.87, 50.26	
Poor or very poor	261 (35.1)	43.92 ± 7.953	42.95, 44.89	

**One-way ANOVA

As Table 5 shows, after adjustment for age and gender, the stepwise multiple regression analysis indicated that the final model contained six significantly predictive variables that accounted for 25.7% of the variance in OHRQoL (F = 29.58, *p* < 0.001). Surveyed elderly participants who were female or had fair or poor self-rated oral health, DMFT scores ≥20, fair or poor self-rated general health, or ≥ 2 teeth with root caries had lower GOHAI scores. In addition, participants who were edentulous had higher GOHAI scores.

**Table 5:** Multiple linear regression model identifying the variables that influenced the GOHAI score

Variable	Coefficient B	95% CI	Standard coefficient	*p*-value
Age	0.880	−0.079, 1.839	0.057	0.072
Gender	− 1.491	− 2.246, − 0.537	−0.098	0.002
Self-rated oral health (reference group: Good or very good)				
Fair	−1.599	−2.874, −0.325	−0.103	0.014
Poor or very poor	− 6.622	− 7.944, − 5.300	−0.415	< 0.001
DMFT (reference group: 0–9)				
≥ 20	−3.227	−4.541, − 2.014	−0.181	< 0.001
Self-rated general health (reference group: Good or very good)				
Fair	−1.960	− 3.316, − 0.804	−0.128	0.001
Poor or very poor	−3.049	− 4.407, − 1.692	−0.171	< 0.001
Number of natural teeth (reference group: ≥25)				
0	3.547	0.869, 6.226	0.092	0.010
Number of teeth with root caries (reference group: 0)				
≥ 2	−1.171	− 2.233, −0.208	−0.072	0.031
Intercept	54.810	52.550, 57.069	–	< 0.001

## Discussion

Compared with data from a decade ago among elderly residents aged 65 to 74 years in Sichuan Province, the mean number of natural teeth (not including third molars) in 2015 to 2016 has increased to 22.19, while the risk of caries continues to rise, resulting in a caries prevalence of 83.2% [17]. Regarding OHRQoL, the mean GOHAI score and its standard deviation of elderly participants in Sichuan Province (48.23, 7.62) indicate that elderly people in Sichuan have relatively better OHRQoL than those in Guangdong Province (45.95, 7.05) and Shandong Province (46.0, 8.5) [15, 16].

Regarding the socio-demographic characteristics, with the exception of gender, none of the factors were related to higher GOHAI scores, as the stepwise multiple regression model showed. Regarding gender, some studies concluded that males had better OHRQoL [21, 22], and one study found the opposite [5], while many other researchers found no association between gender and GOHAI scores [16, 23]. In this study, we found that being female was a strong predictor of lower GOHAI scores, probably because when compared with males, females are more likely to be more unsatisfied with their oral physical function, to have complaints about their discomfort and to have higher expectations regarding their quality of life [22]. In terms of educational level, subjects with higher educational levels may have paid more attention to their oral health and utilized more dental services, therefore leading to a better OHRQoL, which is consistent with studies conducted in Mexico, Germany and Central China [5, 13, 24].

Of the four health-related behaviors variables, brushing habits had a positive influence on the OHRQoL. High-frequency brushing habits, as a basic oral hygiene behavior, tend to lead to healthier oral conditions and better OHRQoL [25]. However, the results related to smoking and drinking habits require further research. The parametric tests indicated that elderly subjects with a history of smoking and drinking had higher GOHAI scores, which is contrary to what one might hypothesize. These results are inconsistent with those of other studies [26, 27] and can perhaps be interpreted in terms of gender differences. In this study, 95.4% of the smokers and 74.65% of the drinkers were male. The Spearman rank correlation test showed correlations between gender and smoking and drinking habits, and the parametric test proved that gender had a significant association with GOHAI scores. Therefore, there may be an overt gender difference that resulted in this unexpected finding.

For oral clinical variables, higher DMFT scores and root caries had a negative impact on OHRQoL. Root caries cause constant tooth sensitivity in response to thermal and chemical stimuli, and compared with coronal caries, root caries are harder to find and restore; therefore, it is not surprising to see this negative impact, which is in line with a survey conducted in America [28]. Regarding the DMFT score, scholars from Mexico and Hong Kong had consistent results, showing that elderly subjects with higher DMFT scores had higher average scores on the GOHAI [9, 14]. For elderly participants aged 65 to 74 years, missing teeth form the majority of the DMFT score; in this study, as Table 4 shows, dentulous participants who had ≥25 natural teeth had higher GOHAI scores than subjects with fewer natural teeth, and at the same time, the multiple linear regression model showed that edentulous status was associated with better OHRQoL, consistent with the finding of ME Northridge et al. [29]. Among the 30 edentulous subjects in the present study, 25 used complete dentures, and the others used informal dentures; parametric tests showed that among denture users, subjects with complete dentures had the highest mean GOHAI score. We could assume compared with dentulous elders, edentulous elders were not affected by the discomfort of their remaining teeth, not having non-functional teeth, or the need to repair lost teeth; furthermore, after their occlusal function was restored with the complete dentures, they might be more receptive to the limitations posed by the dentures [23] and thus more satisfied with their OHRQoL.

Among the 744 participants, only 4.03% of them were edentulous, but 11.29% of them had no occluding tooth pairs, and 40.7% had fewer than nine occluding tooth pairs. Scholars surveyed 1515 elders in southern China and proved that they were more likely to have difficulty chewing hard foods when they had fewer than 8 occluding tooth pairs [30]. The parametric tests used in this study showed that the presence of more functional occluding pairs predicted a higher GOHAI score, suggesting that efforts to maintain occlusal relationships may favorably affect occlusal functions and OHRQoL. Additionally, subjects who need dentures but do not have them had a worse OHRQoL, which is in line with the abovementioned results. Researchers in Korea surveyed 439 elderly patients and proved that satisfaction through the provision of dentures was associated with improved OHRQoL [31], and Brazilian scholars concluded that elderly subjects who needed dental prostheses were more likely to have poor and moderate GOHAI scores [32]. For elderly men with residual roots or missing teeth, functional dentures improve their oral physical function and aesthetics and increased their self-confidence. In contrast, informal dentures often damage adjacent teeth, gingiva and oral mucosa; in this study, participants who used informal dentures had obviously worse OHRQoL, indicating the importance of using formal and functional dentures.

Regarding subjective health conditions, a large number of studies have proved that self-rated oral and general health can explain a large proportion of the variance in GOHAI scores. Japanese scholars surveyed 175 participants and concluded that the mean GOHAI scores were significantly lower for participants with poor perceived oral health or poor perceived general health [12], and many other studies had consistent results [9, 31]. Self-rated oral health is a sophisticated index that relates to diverse factors. When subjects rate their oral health, the different frames of reference for each individual lead to different rating choices. Health behaviors, dental problems, pain, quality of care, tooth loss and social comparisons are the six summary ratings [33], and the GOHAI discriminates between self-perceived oral health and self-perceived general health [14]. Our results reinforce this finding, proving that elderly participants who rated their oral or general health as good had higher GOHAI scores.

However, we must accept that there are limitations in this study. Our stepwise multiple regression analysis results could only account for 25.7% of the variance in OHRQoL. In other words, three fourths of the variance remained unexplained.

## Conclusion

In conclusion, for elders living in Sichuan Province, the explanatory variables of OHRQoL were gender; self-rated oral health; DMFT score; self-rated general health; number of natural teeth; and number of teeth with root caries. Although variables such as educational level, smoking, drinking and brushing habits, number of functional occluding pairs, etc. were related to their OHRQoL, they were not the explanatory variables. To improve the OHRQoL of elders in Sichuan Province, it is important to reduce their high prevalence rates of caries, keep more healthy teeth, improve the cure rate of oral diseases to preserve a healthy mouth.

## Abbreviations

DMFT: Decayed, missing and filled teeth
GOHAI: General Oral Health Assessment Index
LOA: Loss of attachment
OHRQoL: Oral health-related quality of life
